# Skeletal defects and bone metabolism in Noonan, Costello and cardio-facio-cutaneous syndromes

**DOI:** 10.3389/fendo.2023.1231828

**Published:** 2023-10-27

**Authors:** Anna Papadopoulou, Evangelia Bountouvi

**Affiliations:** ^1^ Laboratory of Clinical Biochemistry, University General Hospital “Attikon”, Medical School, National & Kapodistrian University of Athens, Athens, Greece; ^2^ Neonatal Intensive Care Unit, General Hospital “Alexandra”, Athens, Greece

**Keywords:** CFC, bone homeostasis, skeletal dysmorphisms, RAS/MAPK signaling, RASopathies, short stature, Noonan, Costello

## Abstract

Noonan, Costello and Cardio-facio-cutaneous syndromes belong to a group of disorders named RASopathies due to their common pathogenetic origin that lies on the Ras/MAPK signaling pathway. Genetics has eased, at least in part, the distinction of these entities as they are presented with overlapping clinical features which, sometimes, become more pronounced with age. Distinctive face, cardiac and skeletal defects are among the primary abnormalities seen in these patients. Skeletal dysmorphisms range from mild to severe and may include anterior chest wall anomalies, scoliosis, kyphosis, short stature, hand anomalies, muscle weakness, osteopenia or/and osteoporosis. Patients usually have increased serum concentrations of bone resorption markers, while markers of bone formation are within normal range. The causative molecular defects encompass the members of the Ras/MAPK/ERK pathway and the adjacent cascades, important for the maintenance of normal bone homeostasis. It has been suggested that modulation of the expression of specific molecules involved in the processes of bone remodeling may affect the osteogenic fate decision, potentially, bringing out new pharmaceutical targets. Currently, the laboratory imprint of bone metabolism on the clinical picture of the affected individuals is not clear, maybe due to the rarity of these syndromes, the small number of the recruited patients and the methods used for the description of their clinical and biochemical profiles.

## 1 Introduction

Since the first description of nine patients with “pulmonary valve stenosis, small stature, hypertelorism, mild intellectual disability, ptosis, undescended testes, and skeletal malformations” (1), the multiple “faces” of Noonan syndrome(NS) came to light stepwise, thanks to the refined clinical observation and elucidation of the causative molecular pathways. Based on new evidence, the term “RASopathies” has been introduced to describe a group of phenotypically similar congenital anomaly disorders associated with pathogenic variants in genes of the Ras/MAPK signaling pathway, a cascade critical for the regulation of cell proliferation and differentiation (2, 3). Although strict genotype–phenotype correlations are hard to be established, some distinctive features have helped in clinical diagnosis and monitoring (4–7). For example, about half of NS patients have mutations in the *PTPN11* gene, albeit mutations have also been found in other genes involved in the Ras-cascade (8–17). Costello syndrome(CS) has been associated with mutations in *HRAS (* 18), and cardio-facio-cutaneous syndrome(CFCS) with *BRAF, MAP2K1, MAP2K2* and *KRAS (* 11, 19, 20).

Skeletal dysmorphisms, variably and heterogeneously presented, are among the major diagnostic features of RASopathies (6). Prevalent musculoskeletal defects include thoracic wall anomalies, spinal deformities, pes planus, hand anomalies, hip dysplasia, joint laxity or contractures, muscular hypotonia and muscle paucity (21–25). Short stature is also a common clinical sign especially of NS, Costello and CFC syndromes. Notably, growth in NS individuals during childhood and adolescence is often below the 3^rd^ centile (26). Moreover, although still poorly understood, a restricted number of studies have suggested the implication of Ras/MAPK signaling pathway in decreased bone density(BMD) in some RASopathies, -particularly CS (25, 27, 28), NF1 (29–32) and NS (33)- increased bone resorption, as measured by urine degraded crosslinks of mature collagen in NS, Costello and CFC patients (34) and osteoporosis/osteopenia (27, 35–37).

The causative mechanisms behind osseous defects and altered bone metabolism in NS patients remain elusive. The presence of skeletal abnormalities in Ras/MAPK disorders suggest the implication of Ras signal transduction pathway in bone remodeling, while cross-talks with other molecular cascades, such as BMP/Smad/Runx2, may explain the organ-specific pathophysiology (38). The small number of patients included in clinical reports, sometimes without molecular diagnosis of NS, the wide range of patients’ age, the variety of methods and the criteria used for the diagnosis of osteopenia/osteoporosis in children and adults have hampered the deduction of clear inferences so far. Additionally, although skeletal abnormalities are observed in all RASopathies, most focus has been placed on NF1 syndrome. This review summarizes the current knowledge on musculoskeletal phenotypes and biochemical profiles regarding bone metabolism markers seen in NS, CS, CFC syndromes, citing common and distinctive features between these entities, as well as the existing evidence regarding the involvement of the Ras-cascade in bone defects and remodeling.

## 2 Musculoskeletal features and biochemical bone profile

Noonan, Costello and Cardio-facio-cutaneous syndromes are rare disorders, yet the overall prevalence is nearly 1:2,000. Among them, NS has emerged as the more frequent, while CFC and CS constitute ultra-rare diseases with only some hundreds of affected individuals reported worldwide (39–42). The last years, the musculoskeletal profile of NS/CS/CFC has been outlined in the literature, albeit to a lesser degree compared to other pathological aspects of RASopathies (23–25, 28, 34–36, 43, 44). Despite the overlapping phenotypic imprint and the broad clinical variability -even within the same entity-, some recognizable features may orientate the diagnosis to a certain condition and its distinctive underlying genotype. Stature, bone structure and musculoskeletal deformities -further analyzed below- are the basic aspects of osseous phenotype in RASopathies.

### 2.1 Short stature

Short stature is a universal component of Rasopathies. It has been reported in roughly half of NS patients and it is a cardinal feature in CFC (>70%) and CS (>90%) (45–51). Typically, growth parameters at birth are within normal range or even above average. However, during the first years of life postnatal growth failure ensues, leading to a low mean height, further compromised by delayed puberty and an attenuated growth spurt (23, 48). Thus, normative growth charts have been developed for NS (52) and CS (53) patients. Along with hormonal factors, musculoskeletal impairment, gastrointestinal abnormalities and increased resting energy expenditure are also implicated in the pathogenesis of short stature (37, 42, 48, 54–56). The above features are more prevalent and severe in CS and CFC and this partially counts for the more profound height restriction observed compared to NS (25, 36, 45, 57, 58). Notably, progressive musculoskeletal deformities exert a constant negative impact on stature even in adulthood (25, 27, 55, 59). Contrary to CFC and CS, some genotype-phenotype correlations have been suggested for NS. Thus, mutations in *RAF1*, *SHOC2* and *PTPN11* are associated with more severe growth impairment contrary to *SOS1* genotypes (60–62). Of note, *PTPN11* pathogenic variations observed in NS with multiple lentigines(NSML) patients have been linked to lower incidence of short stature (51).

The underlying mechanisms of growth failure seem to be multifactorial and not fully elucidated. As discussed below in the text, molecular defects in components or modulators of the Ras/MAPK pathway have been proposed to induce dysregulation at different levels of the cascade leading to height restriction via diverse mechanisms (48). Hence, consequent dysregulation of GH axis can either lead to GH-deficiency and impaired IGF-1 production (37, 45, 63) or GH-resistance/insensitivity at a postreceptor level (64). Besides, a direct effect of Ras/MAPK pathway on growth plate development has been also demonstrated (65, 66). Exogenous GH has been administrated to overcome the subjacent imbalance in GH-signaling, showing good tolerance in NS and CFC patients and some efficiency mainly during the first two years of regimen (45, 48, 67). Notably, growth hormone therapy has been reported to improve final height in the majority of children with NS (68). Typically, dosage is around 50microgram/kg/day or lower in selected cases (68). Since growth hormone replacement therapy in NS prepubertal years has been demonstrated to be important for the final height, therapy introduction is recommended to initiate at the age 4 in most children or earlier in cases of extreme height restriction. Although a clear connection of this therapeutic intervention with hypertrophic cardiomyopathy or malignancy has not been clearly established, patients’ genotyping is mandatory as specific mutations in *PTPN1* associated to leukemia or mutations in genes linked to the development of hypertrophic cardiomyopathy (e.g., *RIT1*) [reviewed in (68, 69)] are among the exclusion criteria. Notably, in children with mutations known to increase leukemia risk (such as p.Asp61Gly and p.Thr73lle in *PTPN11*), therapy initiation is not recommended before the age of 5 (70). In CS, data are sparse and precautions must be taken due to comorbidities, such as obstructive apnea syndrome and malignancy, apart from the commonly coexisted hypertrophic heart disease (55).

### 2.2 Osteopenia and bone metabolism

Bone mineral density (BMD) measurement, as a marker of bone health, has been used in NS/CS/CFC studies and low values have been documented in all three syndromes (28, 33–35, 37, 43, 68, 71). Short stature has to be taken into consideration at DEXA evaluation –mostly utilized in current studies-, however lower BMD in NS/CFC/CS patients has been also confirmed after comparison to matched control individuals (33, 35, 43). Current data comes from few cohorts, including a limited number of patients, or case series, thus its frequency is rather difficult to be accurately estimated. Evidence from small NS pediatric cohorts underscores the impairment of bone quality and structure. Noordam et al. reported a marginal decrease in BMD of 16 patients with a slight improvement after 2 years of growth hormone replacement treatment (72). Reduced BMD was also documented in 6 of 12 individuals studied by Choudhry et al. (33). Moreover, approximately 15% of the thirty-five patients enrolled by Baldassare et al. showed compromised bone quality as assessed by phalangeal quantitative ultrasound (QUS) (71) while in a recent retrospective study of 35 individuals, axial and appendicular bone mass were reduced compared to the general population (37). Data in NS adult patients are even scantier. Five over thirty-five individuals have been reported with osteopenia or osteoporosis and two over twenty-six with osteoporosis by Smpokou and Reinker et al, respectively (36, 73). Bone metabolic disease in NS has been supported to be partly primary resulting from an imbalance between osteoclast and osteoblast activity due to RAS/ERK hyperactivation, as discussed below (33, 37, 71). Notably, recent results from experiments in transgenic NS mice revealed an essential role of SHP2 in promoting osteoblastic differentiation and suppressing osteoclastogenesis (74, 75). Moreover, BMD was shown to correlate with decreased muscle mass and low IGF-1 levels (37), while low gonadal steroids also supported to contribute to the final bone phenotype (37, 48, 68, 76).

In CS, osteopenia and osteoporosis have been described as a common feature in a few reports/case series, presenting an increasing incidence with age, which is feasible since bone pathology evolves over time (28, 35, 77). Stevenson et al. described low BMD in seven CS patients (34). Detweiler et al. reported a frequency of 41% (N=34) and 6% (N=33) for osteopenia and osteoporosis, respectively, on a cohort of 43 CS individuals (26 pediatric and 17 adolescent/adult patients) (77). In a series of adult patients, BMD was detected to be impaired in all eight individuals assessed (2 with osteopenia and 6 with osteoporosis). Three of them were symptomatic suggesting clinical significance of osteoporosis in CS (27). Supporting this notion, two of the three infants reported in a small case series suffered by generalized osteoporosis (78). Leoni et al. in 2014 demonstrated reduced BMD in 9 CS individuals compared to age-matched controls (from 5 to 29 years of age) (28). Subsequently, the same group confirmed these results in an extended cohort of 16 individuals over a 4-year follow up study (35). Cakir et al. reported a CS adult patient with a parathyroid adenoma, suffering from osteoporosis since adolescence (79). Many factors have been supported to contribute to final bone phenotype such as decreased mobility, reduced peak bone mass, muscle impairment and severe hypogonadism in some patients (27, 44, 48). The last years, the notion that BMD is also an intrinsic feature of CS has gained ground. Increased RAS/ERK signaling in CS has been proposed to induce osteoclastic hyperactivity and increased bone resorption as supported by studies of bone resorption markers (28, 34).

Data on prevalence of osteopenia or osteoporosis in CFC are sparser (43, 48). Apart from one patient reported with slightly decreased BMD and biochemical evidence of elevated markers of bone resorption (34), Chen et al. recently described one 10 year-old girl suffering from osteoporosis and multiple musculoskeletal deformities in a small series of 3 *MAP2K1* CFC patients (80). The first systematic cohort is a recent study of 14 patients who displayed low BMD compared to healthy age-matched controls, despite proper cholecalciferol supplementation. However, only one child met the osteoporosis diagnostic criteria (43).

Overall, the risk of fracture is currently difficult to be determined, yet longitudinal BMD monitoring is recommended. Apart from bone fragility, decreased BMD has been also associated with pain (48, 58), low physical activity (48), low muscle mass, depressed IGF-1 levels (37) and inferior life quality (25, 58). Despite the fact that evidence is lacking pertaining to CFC, upregulated function of RAS/MAPK cascade has also been implied in impaired bone metabolism. Notably, mild increased levels of alkaline phosphatase(ALP) have been reported in CFC patients (25). Besides genetic background, other factors also contribute to bone homeostasis alteration such as lack of autonomous deambulation, sedentary lifestyle and common used medications in CFC (antiepileptics, proton pump inhibitors).

Although pathogenetic mechanisms and association with genetic deficits are not fully understood, emerging data from different groups have underscored the essential role of Ras/MAPK signaling pathway in bone metabolism (23–25, 44, 48). The imbalance between osteoclastic and osteoblastic activity as a result of Ras/MAPK signaling deviation have been proposed to be fundamental in osseous pathophysiology (48). Indeed, preclinical and experimental data have revealed up-regulation of osteoclasts’ development (65, 81), function and survival and, on the other hand, impaired growth and differentiation of osteoblasts along with decreased osteoblasts’ activity (23, 24, 71, 82). The above hypothesis is further supported by biochemical data from NS/CFC/CS individuals; markers of bone resorption such as urine excretion of pyridinium crosslinks (28, 34), and PTH (48) have been elevated while decreased levels of calcitonin –a biomarker of osseous formation and osteoblastic activity- has been determined (48). Constitutional decrease of 25-Vitamin D has been constantly described in all 3 entities, yet no specific correlation with the low BMD has been confirmed (33, 37, 43, 71). Noteworthy, reduced BMD in patients exhibiting a rather normal bone metabolism profile have been also reported (25, 28, 33). Of note, in three different NS pediatric cohorts investigated, all showed normal mean serum calcium, phosphate and ALP concentrations (33, 37, 71). These observations underline the fact that bone homeostasis is a dynamic biological process modulated by complex physiological and environmental influences. Therefore, apart from the intrinsic bone pathology in RASopathies, various environmental and secondary to diseases’ morbidities factors converge into the final bone phenotype. Collectively, deviation of Ras/MAPK pathway, progressive bone deformities, limitations in physical activity, hormonal disturbances such as hypogonadism and perturbed IGF-1 activity, elevated inflammatory cytokines, sun exposure, musculature impairment and medications such as antiepileptics, proton pump inhibitors and corticosteroids are implicated in the altered bone homeostasis observed in RASopathies (23, 44, 48, 83).

### 2.3 Skeletal deformities

Musculoskeletal deformities have been described as another overlapping component of RASopathy nosologies. As mentioned above, the list of skeletal manifestations, and the incidence of each deformity is rather difficult to be accurately estimated due to the rarity of the disorders, the broad clinical spectrum, the dynamic process of bone development and the heterogeneity among studies focusing on different ages–mainly pediatric-and on diverse diseases’ aspects. Thus, many skeletal findings remain underreported. Although all RASopathies include skeletal defects, CFC and CS have been documented to be characterized by a distinctive osseous phenotype and severe skeletal involvement, especially compared to milder bone manifestations reported in NS patients (23–25). The main skeletal characteristics are presented below and summarized in **Table 1**.

**Table 1:** Main skeletal clinical characteristics of Noonan, Costello and Cardio-facio-cutaneous syndromes.

	Noonan (%)	Costello (%)	Cardio-facio-cutaneous (%)
Selected skeletal findings			
Cranium			
Craniofacial dysmorphisms	+++++ (100)	+++++ (100)	+++++ (100)
Macrocephaly (relative or absolute)	+ (2)	++++ (93)	++++ (86-97)
Craniosynostosis	+ (9-10)	++ (33)	One case reported
Distinctive signs that may help to differential diagnosis	Hypertelorism, down-slanting palpebral fissures, ptosis, micrognathia, triangular face appearance, shorten neck, low set ears	Macrocephaly, bi-temporal narrowing, coarse facial features, large mouth with thick lips, low set ears, depressed nasal bridge	Macrocephaly with bi-temporal narrowing, highly arched palate appearance of open bite
Axial skeleton			
Pectus excavatum carinatum	++++ (28-95)	+++ (6-62)	+++ (12-65)
Scoliosis	++ (13-30)	+++ (17-63)	++ (25-35)
Kyphosis	+	+++ (17-58)	++ (18-23)
Appendicular skeleton			
Cubitus Valgus	+++ (45-50)	++ (29-50)	++(24)
Ulnar deviation of wrist		+++ (22-82)	++ (35)
Laxity of small joints	++++	++++ (41-100)	++ (12-65)
Contractures of large joints Elbow Knees		+++ (50-63) ++ (18-33)	++ (12-30) ++ (22-35)
Heelcord contructures		+++ (40-82)	+ (6)
Pes planus	+	+++ (35-53)	++(18-62)
Pes cavus		++ (24-40)	+ (6)
Hip dysplasia		+++ (15-89)	++ (16-27)
Distinctive signs that may help to differential diagnosis	Cubitus Valgus with brachydactyly	broad, short hand, flexed wrists/elbows and ulnar deviation of fingers (80), tight Achilles tendons	higher incidence of lower extremities pathology and hip dysplasia

The small sample size, the wide age range, and the variety of methods used in a restricted number of clinical reports lead to a large percentage range of developing symptoms. Therefore, we also use “+” symbol in order to roughly present a magnitude of the estimated prevalence, taking into account the number of reported cases with a particular symptom/study.

Noonan (4, 24, 51, 87, 97): CS (21, 24, 25, 27, 36, 42, 77, 85, 102): CFC (24, 25, 36, 49, 84):.

Craniofacial features are part of developmental anomalies syndromes and in RASopathies include, among others, relative or absolute macrocephaly with bitemporal constriction, prominent forehead, highly arched palate and dental abnormalities in all three disorders (40, 84, 85). Moreover, pertaining to NS, micrognathia and a triangular face appearance is a distinctive sign, while despite the large appearance of cranium (4, 86, 87), head circumference in adulthood does not differ significantly compared to general population (88). The vast majority of CFC and CS patients do present with macrocephaly (>85% and 93%, respectively) and bi-temporal narrowing. Large mouth with thick lips is a distinctive sign in CS, while appearance of open mouth, partially attributed to the existence of highly arched palate(up to 80%), which is more common in *BRAF* mutated CFC individuals (41, 84, 89). Recently, RASopathies have been associated to an increased risk of craniosynostosis, expanding the clinical phenotype. In a latest study, it has been suggested that about 33% of CFC and 9% of NS individuals may suffer from craniosynostosis (90). The patients reported owed mutations in *BRAF, SHOC2, PTPN11* and *KRAS* genes, with the latter proposed to be correlated to more severe craniosynostosis (90–94). Craniosynostosis has been also described in a CS patient harboring a duplication in *HRAS* gene (95). It is worth to mention that in a recent retrospective study late onset synostosis was prominent (92), underscoring both the need for long-term monitoring and the record of this clinical sign.

Deformities of axial skeleton are cardinal features in all three entities (40). Chest wall anomalies, such as pectus excavatum and/or carinatum combined with a broad thorax is distinctive in NS (up to 95%) (23, 24). Similarly, the above pectus abnormalities are common both in CFC and CS and have been reported in up to two-thirds of affected individuals (25, 50, 96). Scoliosis/kyphosis are other universal manifestations reported roughly in 15% of NS (86, 87, 97), 20-35% of CFC and from 17% to more than half of CS individuals (36, 77, 98). Back and chronic pain, especially in adulthood, are more frequently observed in NS patients compared to CS/CFC patients, while kyphosis is not (36, 58).Of note, vertebral reversal with lumbar kyphosis and thoracic lordosis has been described in CS (77). Moreover, in CS individuals, kyphoscoliosis has been recently demonstrated to be slowly progressive in the absence of structural neurological deformities (98).

Appendicular skeleton is also affected (23, 40). Cubitus valgus (ulnar deviation of the elbow) together with brachydactyly is the most common deformity of the upper extremities, presented in nearly half of NS affected individuals (23, 24). Furthermore, CS patients present a more severe abnormal posture including broad, short hand, flexed wrists/elbows and ulnar deviation of fingers. This characteristic posture affects nearly 80% of CS patients, is highly suggestive of the disorder and is partially attributed to laxity of small joints (21, 36). Contrary to hyperextensibility of small joints, large joints are compromised by contractures. Other characteristic features in CS are pes planus, tight Achilles tendons -that even in childhood often require surgical therapy, and hip dysplasia (55). The latter affects 15-89% CS individuals (21, 25, 77) and whereas ligamentous laxity and hypotonia have been proposed as main etiologies of early presented bilateral dysplasia, underlying mechanism of late unilateral onset forms remains elusive (55, 56). All above upper and lower limbs deformities have been also reported in CFC (89). Signs that may differentiate CFC are the higher incidence of lower extremities pathology, such as short and broad feet, knee contractures(~25%), pes planus/cavus (up to two thirds) and hip dysplasia(17-27%) (25, 36, 41, 49). What is more, two case-descriptions of CFC patients have reported on skeletal manifestations, previously unreported; symmetric polyostotic fibrous dysplasia of proximal humerus (99) and symmetrical polyarthritis affecting upper and lower extremities (100). Given the rarity of RASopathies even one case can significantly alter statistics and focus attention on phenotypes that may be under recognized.

Few genotype-phenotype correlations have been proposed. CS-patients harboring the rare mutation *Gly13Cys* have been demonstrated to exhibit a milder orthopedic phenotype. On contrary, CFC-patients with genetic defects in *MAP2K1/2* are presented with more severe osseous manifestations, higher incidence of multiple joint contractures and scoliosis (25).

Since bone homeostasis is a dynamic and multifactorial, process longitudinal monitoring of skeletal manifestations is of high importance as a progressive and worsening pattern is displayed especially in CS and CFC individuals. Hence, compromised independent walking, surgery interventions, limited physical activity are often an issue (25, 55, 89). It is also noteworthy that along with intrinsic bone pathology in RASopathies other factors exert a significant role in final bone phenotype. More precise, some of bones’ features are secondary to other comorbidities such as neurological impairment, intellectual disability, reduced physical activity, hypotonia and lacking musculature (25, 44, 58, 83).

Indeed, generalized muscular hypotonia of varying severity and hypotrophy is a rather consistent feature. Abnormalities on muscle biopsies of CFC/CS individuals, along with data derived from *in vitro* studies and recent experiments in transgenic mouse models provide further support to the presence of intrinsic myopathy in CS/CFC syndromes (6, 23, 44, 101–103). As a pivotal role of RAS/MAPK signaling cascade in modulation of skeletal myogenesis has been demonstrated, it has been speculated that paucity of muscle fiber and hypotonia could be attributed to impaired myoblast differentiation during the early stages of muscular development (25).

## 3 Ras/MAPK cascade and bone defects

Research on the molecular etiology of bone defects of NS and other RASopathies focuses mainly on the members of the Ras/MAPK/ERK pathway and, their synergy with other cascades, important for the maintenance of normal bone homeostasis (104) such as Wnt-frizzled/β-catenin, BMP/SMAD/Runx2 and PI3K/AKT (38, 105). Although the effects of the Ras-signaling on osteogenesis are not well defined, emerging evidence suggest that Ras/MAPK pathway is a positive regulator of mesenchymal progenitor cells differentiation into osteoblasts and of osteoblast proliferation during skeletal development (36, 106–108). Recently, it has been demonstrated that inactivation of ERK in osteoprogenitors at an early postnatal stage of skeletal development in mice results in severe bone loss and cleidocranial dysplasia phenotypes (109).

The driver molecules of the Ras transduction signaling are small GTPases of the Ras family(H-ras, K-ras, N-ras), differentially stimulated by diverse upstream effectors, that mediate the translation of extracellular signals in specific genes through downstream cascades such as the well described MAPK axis (Raf-1/mitogen-activated, ERK-activating kinase) (3, 110). Activating mutations in any component of this pathway may result in increased MAPK activity, unrestrained cell proliferation and deviation from the physiological pattern of differentiation.

Among the Ras upstream effectors, SHP2, a protein tyrosine phosphatase encoded by the *PTPN11* gene is involved in the regulation of proliferation, differentiation and recruitment of mesenchymal stem cells (MSCs) and of neural crest cells (NCCs) in response to multiple growth factors such as epidermal growth factor (EGF) and platelet derived growth factor (PDGF) via the MAPK pathway (111, 112). It has been shown that MSCs SHP2-deficient mice display multiple abnormal skeletal phenotypes, including postnatal growth retardation, shortened and malformed limbs and pectus excavatum/carinatum (113). Additionally, mice in which SHP2 is deleted in chondrocytes at 4 weeks after birth have scoliosis and kyphosis, a phenotype not seen when the deletion is induced in adult mice (114). In a subsequent study, the lack of SHP2 from hypertrophic chondrocytes showed a significantly decreased number of osteoblasts in the cancellous bone and repressed osteogenic gene expression suggesting a potential role for SHP2 in the regulation of hypertrophic chondrocyte transdifferentiation (115). The significantly diverse phenotypes of the mice lacking SHP2 in proliferating and hypertrophic chondrocytes indicate a developmental stage-specific role for SHP2 in chondrogenesis and bone mineral homeostasis (114), reviewed in (75).

As mentioned earlier, short stature is a common feature of NS. It has been shown that mice bearing *PTPN11* gain of function mutations are smaller with increased skull size and hypertelorism (116), while the growth plate length is reduced mainly due to the decrease of the hypertrophic zone (66). NS-causing SHP2 mutants inhibit GH-induced IGF1 release through Ras/MAPK hyperactivation, a mechanism that may lead to growth retardation, suggesting that the short stature seen in *PTPN11* NS patients may be alleviated by modulating the activation of this cascade (117).

Activated Ras proteins can also promote or inhibit a number of signaling cascades such as BMP/SMAD/Runx2 and modulate osteogenic gene expression and differentiation. BMP-signaling pathway enhances osteoblast differentiation through SMAD1/5/8, Runx2 and osterix activation (109, 118). *In vitro* studies on induced pluripotent stem cells from an individual with CFCS that were differentiated into MSC showed enhanced MAPK/ERK activation and SMAD3 hyperactivation leading to an altered osteogenic differentiation with reduced ALP activity and impaired mineralization (82). Similarly, MSC from CS patients showed poor differentiation toward the osteoblastic lineage *in vitro*, with low ALP and poor mineralization (119). The Wnt/β-catenin (120), PI3K/AKT (121) as well as other pathways involved in bone remodeling are implicated in cross-talk with Ras/MAPK cascade, however the interaction between them is not clear. Some months ago, Yang et al. attributed a new crucial role of KRAS in regulating extracellular matrix accumulation for optimal osteogenesis. It has been shown that stem cells coordinate the expression of small GTPases to modulate autophagy, thereby promoting osteogenic differentiation (122).

The striking complexity of the RAS signaling cascade, the plurality of its molecular components and the interference with various epigenetic factors complicate the understanding of the underlying mechanisms, offering a challenging research field. In this direction, genetic or pharmacologic inhibition of SHP2 has emerged as a critical node for therapeutic applications. Although various SHP2 competitive inhibitors have been reported, only a few allosteric SHP2 inhibitors have been developed and progressed in clinical trials [for detailed review see (75)]. These allosteric SHP2 inhibitors have been extensively studied in cancer (123) and its introduction to other human genetic diseases and musculoskeletal pathologies is currently under investigation (124, 125). Furthermore, a recent study in CS Hras G12V mouse model demonstrated that inhibition of RAS/MAPK signaling pathway using MEK-inhibitor rescued myopathy phenotype both *in vitro* and *in vivo* (126). In accordance, Nandi et al. using the same mouse model revealed that mutated Hras contributed to increased osteoclastogenesis, which was reduced by *in vitro* treatment with MEK and PI3K inhibitors (127). The above results were further supported by another study on an activating Braf ^L597V^ CFC mouse model. In particular, Ras/MAPK dysregulation during development inhibited myofiber differentiation which was rescued by MEK inhibition (102). What is more, trametinib, a highly selective reversible allosteric inhibitor of MEK1/2 was shown to restrict myocardial hypertrophy in NS *RIT1* mutants (128). Of note, currently the effects of gemline mutations on energy metabolism and mitochondria is being investigated (129). A recent review in multiple Hras CS models highlighted the importance of bioenergetics and mitochondrial proteostasis in heart and skeletal muscles and proposed that CS patients may benefit from regimen with mitochondrial modulators (130). Certainly, high quality clinical studies are warranted to shed light on various aspects and limitations which accompany the novel targeted therapies (23, 75).

## 4 Conclusions

As evidenced there is significant overlap in addition to discordance of the musculoskeletal phenotypes between Noonan, Costello and CFC syndromes. The rarity of the syndromes and the wide range of patients’ age make it difficult to draw safe conclusions in order to clearly define the clinical and biochemical patients’ profile. The clinical complexity reflects the interactions of an extensive molecular network, with the Ras cascade as a starting point and driver. However, the dissociation of some skeletal manifestations (for example chest deformities) from the Ras/MAPK effect on both osteoclastic and osteoblastic cells suggests a possible impact of this cascade on different cell lines interfering with musculoskeletal development. The elucidation of the biological and genetic background of these diseases may reveal novel target therapies not only for musculoskeletal phenotypes but also for other RASopathies’ clinical manifestations, as well as for different entities, such as cancer and autoimmune disorders.

## Author contributions

AP and EB contributed to the conception, design, drafting of the work and revised it critically for important intellectual content. All authors contributed to the article and approved the submitted version.
